# Polycystic ovary syndrome and risk factors for gestational diabetes

**DOI:** 10.1530/EC-18-0076

**Published:** 2018-05-31

**Authors:** Sanna Mustaniemi, Marja Vääräsmäki, Johan G Eriksson, Mika Gissler, Hannele Laivuori, Hilkka Ijäs, Aini Bloigu, Eero Kajantie, Laure Morin-Papunen

**Affiliations:** 1 Public Health Promotion UnitNational Institute for Health and Welfare, Helsinki, Finland; 2 PEDEGO Research UnitMRC Oulu, Oulu University Hospital and University of Oulu, Oulu, Finland; 3 Department of General Practice and Primary Health CareUniversity of Helsinki and Helsinki University Hospital, Helsinki, Finland; 4 Folkhälsan Research CentreHelsinki, Finland; 5 Information Services DepartmentNational Institute for Health and Welfare, Helsinki, Finland; 6 Division of Family MedicineDepartment of Neurobiology, Care Sciences and Society, Karolinska Institute, Stockholm, Sweden; 7 Medical and Clinical GeneticsUniversity of Helsinki and Helsinki University Hospital, Helsinki, Finland; 8 Department of Obstetrics and GynaecologyTampere University Hospital, Tampere, Finland; 9 Faculty of Medicine and Life SciencesUniversity of Tampere, Tampere, Finland; 10 Institute for Molecular Medicine FinlandHelsinki Institute of Life Science, University of Helsinki, Helsinki, Finland; 11 Children’s HospitalUniversity of Helsinki and Helsinki University Hospital, Helsinki, Finland

**Keywords:** gestational diabetes mellitus, polycystic ovary syndrome, obesity, BMI, family history of diabetes

## Abstract

**Objective:**

To study the roles of self-reported symptoms and/or prior diagnosis of polycystic ovary syndrome (PCOS) and other potential risk factors for gestational diabetes mellitus (GDM) and to clarify whether the screening of GDM in early pregnancy is beneficial for all women with PCOS.

**Design:**

The FinnGeDi multicentre case-control study including 1146 women with singleton pregnancies diagnosed with GDM and 1066 non-diabetic pregnant women. There were 174 women with PCOS (symptoms and/or diagnosis self-reported by a questionnaire) and 1767 women without PCOS (data missing for 271).

**Methods:**

The study population (*N* = 1941) was divided into four subgroups: GDM + PCOS (*N* = 105), GDM + non-PCOS (*N* = 909), non-GDM + PCOS (*N* = 69), and controls (*N* = 858). The participants’ characteristics and their parents’ medical histories were compared.

**Results:**

The prevalence of PCOS was 10.4% among GDM women and 7.4% among non-diabetics (odds ratios (OR) 1.44, 95% CI: 1.05–1.97), but PCOS was not an independent risk for GDM after adjustments for participants’ age and pre-pregnancy BMI (OR 1.07, 95% CI: 0.74–1.54). In a multivariate logistic regression analysis, the most significant parameters associated with GDM were overweight, obesity, age ≥35 years, participant’s mother’s history of GDM, either parent’s history of type 2 diabetes (T2D) and participant’s own preterm birth.

**Conclusions:**

The increased risk of GDM in women with PCOS was related to obesity and increased maternal age rather than to PCOS itself, suggesting that routine early screening of GDM in PCOS women without other risk factors should be reconsidered. Instead, family history of GDM/T2D and own preterm birth were independent risk factors for GDM.

## Introduction

Gestational diabetes mellitus (GDM) and polycystic ovary syndrome (PCOS) are the most common endocrine disorders in women of reproductive age. The prevalence of GDM varies from 9% to 25% and the prevalence of PCOS varies from 5% to 15%, depending on the study populations and the diagnostic criteria applied (1, 2, 3, 4, 5). Both disorders are associated with insulin resistance and overweight/obesity (6, 7). Also, genetic factors play a significant role in both conditions (8, 9).

GDM and PCOS can be early signs of an increased risk of manifest diseases related to insulin resistance, such as type 2 diabetes (T2D), and both disorders are also connected with cardiovascular risk factors, such as metabolic syndrome, hypertension and dyslipidaemia (1, 10, 11, 12, 13, 14). While similar metabolic risks are associated with these conditions, the independent role of PCOS and the roles of shared risk factors for GDM are still unclear. Several studies have shown that PCOS increases the risk for GDM independent of the degree of adiposity (2, 15, 16, 17, 18). Mikola *et al*. found that PCOS independently increased the risk of GDM, but that overweight was the strongest predictor for GDM (19). However, another study reported that non-obese women with PCOS were not at risk for GDM (20). Also, two studies including participants matched for BMI and age did not report increased risk for GDM in women with PCOS (21, 22). Despite these inconsistent data, in Finland, PCOS is considered an independent indication for an early oral glucose tolerance test (OGTT) during pregnancy, regardless of BMI, according to the 2008 National Current Care Guidelines (23). In the present study, we aimed to test the validity of this practice further and to clarify the role of PCOS as a risk factor for GDM.

In this case–control setting, our first aim was, therefore, to investigate whether PCOS is an independent risk factor for GDM. This information is important to clarify whether the performance of an early OGTT is necessary for all pregnant women with PCOS regardless of their BMI. Second, we wanted to investigate the role of other shared risk factors for PCOS and GDM, such as overweight or obesity, the woman’s own birth data and current characteristics and her parents’ medical histories. To clarify these issues, we divided the study population into four subgroups according to GDM and PCOS status. We hypothesised that participants displaying both disorders would share more risk factors compared to controls or those with only one disorder.

## Subjects and methods

### Participants

**Figure 1:**
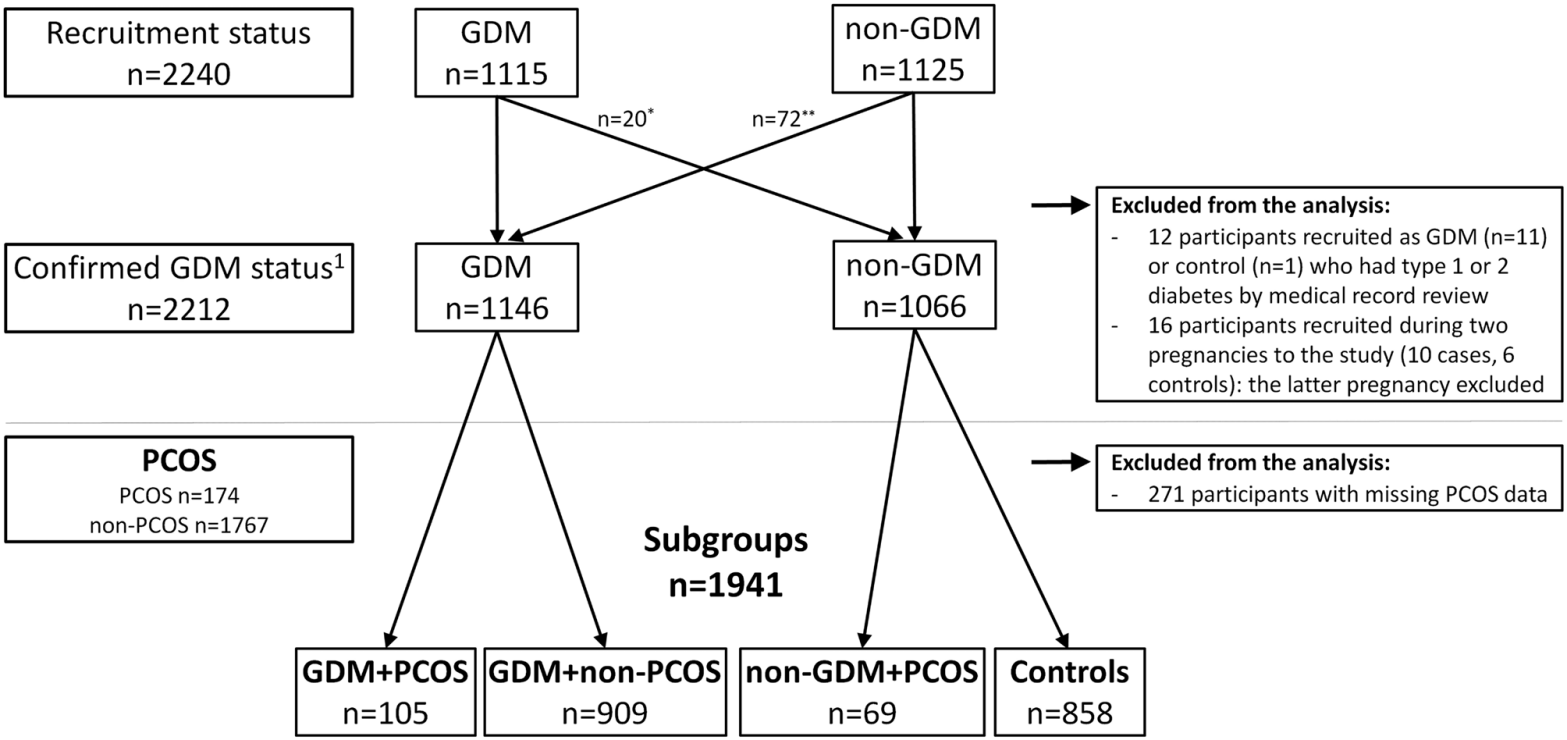
Flow chart of study population. GDM, gestational diabetes mellitus; PCOS, polycystic ovary syndrome. *20 women recruited as GDM cases but confirmed by medical record review to have normal glucose tolerance during pregnancy. **72 women recruited as a control fulfilled the criteria for GDM by medical record review. ^1^Confirmed status of participant, with diagnosis of GDM based on oral glucose tolerance test result(s) (*n* = 1122) or self-monitoring (*n* = 24) and the absence of type 1 or 2 diabetes as indicated by medical record review.

The Finnish Gestational Diabetes Study (FinnGeDi study) aims to study genetic and environmental factors linked to the development of GDM and has two arms: a register-based (5, 24) and a clinical genetic arm. The present case–control study was based on the clinical genetic arm, which was piloted at Oulu University hospital (tertiary-level) in February 2009 and extended after 3 months to six other hospitals (Helsinki and Uusimaa, Central Finland, Satakunta, Southern Ostrobothnia, Southern Karelia and Kainuu), each serving a geographically based catchment area. Recruitment ended in December 2012. Women with GDM and a singleton pregnancy (*N* = 1115) were recruited in delivery units before delivery, and the next consenting mother without GDM (*N* = 1125) giving birth in the same hospital was recruited as a control. The spouses and new-borns of both GDM and non-diabetic mothers were also included in the study. The GDM status of all participants was confirmed by their medical records: 12 women with pre-pregnancy diabetes were excluded. Sixteen women were recruited during two pregnancies, and their latter pregnancy was excluded. In total, 1146 women fulfilled the GDM criteria, and 1066 women were defined as non-diabetic controls (Fig. 1).

### Clinical data

After recruitment, the participants were asked to complete a detailed questionnaire about their medical and family history and lifestyle. This questionnaire also included questions on oligomenorrhoea, the presence of hirsutism and prior diagnosis of PCOS. In total, 1030 (89.9%) GDM and 935 (87.7%) non-diabetic women returned the questionnaire. Comprehensive, detailed data on pregnancy and delivery were collected from the hospital and maternal welfare clinic records by trained research nurses and medical students. Clinical data from the questionnaire and medical records were supplemented by individually linked register data obtained from the Finnish Medical Birth Register (MBR). In Finland, all live births and stillbirths of infants weighing ≥500 g or having a gestational age of ≥22 weeks are reported to the MBR. A structured form is completed by the delivery hospital within one week after delivery. The MBR includes detailed information about the pregnancy, delivery and perinatal health of the new-borns until the age of seven days.

### Definitions

#### Women with GDM

Gestational diabetes was diagnosed by a 2-h 75 g OGTT after overnight fasting. According to the 2008 National Current Care Guidelines, OGTT was recommended for every pregnant woman, excluding women with very low risk for GDM: (1) primiparous women of normal weight (BMI <25 kg/m^2^) who were under 25 years of age and had no family history of diabetes, and (2) multiparous women of normal weight (BMI <25 kg/m^2^) who were under 40 years of age and had no history of GDM or macrosomic births (birth weight over 4500 g) (23).

OGTT was mainly performed between the 24th and 28th weeks of pregnancy and was recommended between the 12th and 16th gestational weeks in cases with a high risk of GDM (prior GDM, BMI ≥35 kg/m^2^, glucosuria, family history of T2D or PCOS). Of note, in Finland, PCOS is an independent indication for early OGTT during pregnancy. If the first OGTT was normal, it was repeated between the 24th and 28th weeks of gestation. The cut-off values for plasma glucose concentrations were ≥5.3 mmol/L after fasting, ≥10.0 mmol/L at 1 h and ≥8.6 mmol/L at 2 h after glucose intake. A diagnosis was made if one or more values in the OGTT were abnormal (23). Additionally, GDM diagnosis was based on glucose self-monitoring for 24 participants. Of these participants, 20 (83.3%) started early self-monitoring because of a previous history of GDM, and four (16.7%) because of unsuccessful OGTT testing. All of them had blood glucose values repeatedly over the target range (fasting glucose ≥5.5 mmol/L and 1 h after meal ≥7.8 mmol/L). Of these participants, 13 (54.2%) were treated with insulin, ten with diet (41.7%) and one (4.2%) with metformin.

#### Women with self-reported symptoms and/or prior diagnosis of PCOS

Because the study was performed at the time of delivery, the definition of PCOS was based on a self-reported questionnaire that included a question on whether the participant had been diagnosed with PCOS and questions regarding excessive body hair and oligomenorrhoea before pregnancy. The criteria for oligomenorrhoea (‘Menstrual cycle often (more than twice a year) more than 35 days without hormonal contraception’) had been validated in our previous studies performed in a large Finnish population-based follow-up cohort (25, 26). Additionally, women with irregular menses (*N* = 97), defined as a difference frequently exceeding 7 days between the longest and shortest menstrual cycles, were included in the oligomenorrhoea group. Hirsutism was assessed if a woman reported excessive body hair or removed facial hair at least four times per month. Again, the validity of this questionnaire to detect women with typical endocrine characteristics of PCOS has been shown in our previous studies (25, 26).

There were 521 (26.8%) participants with oligomenorrhoea or irregular menses, 207 (10.7%) with hirsutism and 87 (4.5%) with both a menstrual disorder and hirsutism. Also, 124 (6.4%) participants reported a prior PCOS diagnosis and 37 (1.9%) reported both a prior diagnosis and two symptoms. Hence, in total, 174 participants with both symptoms and/or prior diagnosis were considered to have PCOS. The controls for the PCOS group included the 1767 participants without two symptoms and without a diagnosis of PCOS. The participants with missing data regarding PCOS symptoms were excluded from the study (*N* = 271).

The study population (*N* = 1941) was then divided into four subgroups: GDM + PCOS (*N* = 105), GDM + non-PCOS (*N* = 909), non-GDM + PCOS (*N* = 69) and controls (*N* = 858) (Fig. 1). Women with GDM, PCOS or both were compared with controls.

### Measures

The questionnaire obtained the participant’s birth weight and gestational age at birth. Preterm birth was defined as gestational age <37 + 0 weeks. Two participants were excluded from the birth weight analyses because they reported abnormally high birth weights for the length of gestation (<37 gestational weeks with >3 s.d.s of birth weight, according to Finnish standards) (27). Small-for-gestational age (SGA) participants were defined as those with a birth weight of <−2 s.d.s and large-for-gestational-age participants as those with a birth weight of >+2 s.d.s. BMI was calculated from participants’ pre-pregnancy height and weight (kg/m^2^), which were obtained from maternity welfare clinic records. BMIs between 18.5 kg/m^2^ and 24.9 kg/m^2^ were classified as normal, those between 25.0 kg/m^2^ and 29.9 kg/m^2^ as overweight and those of 30.0 kg/m^2^ or more as obese (28).

The educational attainment of each participant was obtained from the questionnaire and categorised as basic or less, secondary, lower-level tertiary or upper-level tertiary. When education was not reported (three cases), it was deduced based on the MBR data about the mother’s occupation during pregnancy. Data about smoking before pregnancy were obtained from the questionnaire and data about smoking during pregnancy were obtained from the MBR. Information on participants’ previous pregnancies was obtained from the MBR and questionnaire. Participants’ parents’ education and medical histories, including histories of diabetes and cardiovascular morbidity, were taken from the questionnaire.

### Statistical methods

Data were analysed with SPSS 22.0 (IBM SPSS Statistics for Macintosh, version 22.0. Armonk, NY, USA: IBM Corp.). The subgroups were dummy-coded for statistical analysis, with controls used as references. One-way ANOVA was used for continuous variables and chi-square tests for categorical variables. Continuous variables are reported as mean ± s.d. and categorical variables are reported as frequencies (%). Normal probability plots and visual inspection of histograms were used to assess normality and no transformations were considered necessary. Statistical significance was set at a two-sided *P* value of <0.05. Linear regression (mean differences with 95% CI) was used for continuous variables, and logistic regression (odds ratios (ORs) with 95% CI) was used for categorical variables. Multiple regression models were used. The first model was unadjusted (crude). The effect of adding each variable in sequence was evaluated before fitting the final model. The final model was adjusted for participant’s parity, smoking during pregnancy, educational attainment, delivery hospital, age and pre-pregnancy BMI. Interactions with PCOS and the covariates were tested by adding the product term between the two variables of interest in the regression model to the variables of interest and were found to be non-significant.

#### Covariates

Covariates in this study were participant’s age, pre-pregnancy BMI, parity, educational attainment, smoking during pregnancy and delivery hospital. Participant’s age, pre-pregnancy BMI and parity were included because these potential confounding factors may reflect the course of pregnancy. Educational attainment was used as an indicator of socio-economic status (SES) since there is evidence that low childhood SES may be associated with PCOS (29). There may also be an association between smoking during pregnancy and GDM (30). The delivery hospital was observed to minimise the potential risk that local practices could differ between hospitals. When the present characteristics of participants were studied, these covariates acted as potential confounders. When participants’ characteristics at birth and medical histories of participants’ parents were assessed, the covariates were potential mediators.

### Ethical approval

The study protocol was approved by the Ethics Committee of the Northern Ostrobothnia Hospital District in 2009. Each participant gave written informed consent after full explanation of the purpose and nature of all procedures used. The National Institute for Health and Welfare (THL) permitted the use of MBR data in this study.

## Results

**Figure 2:**
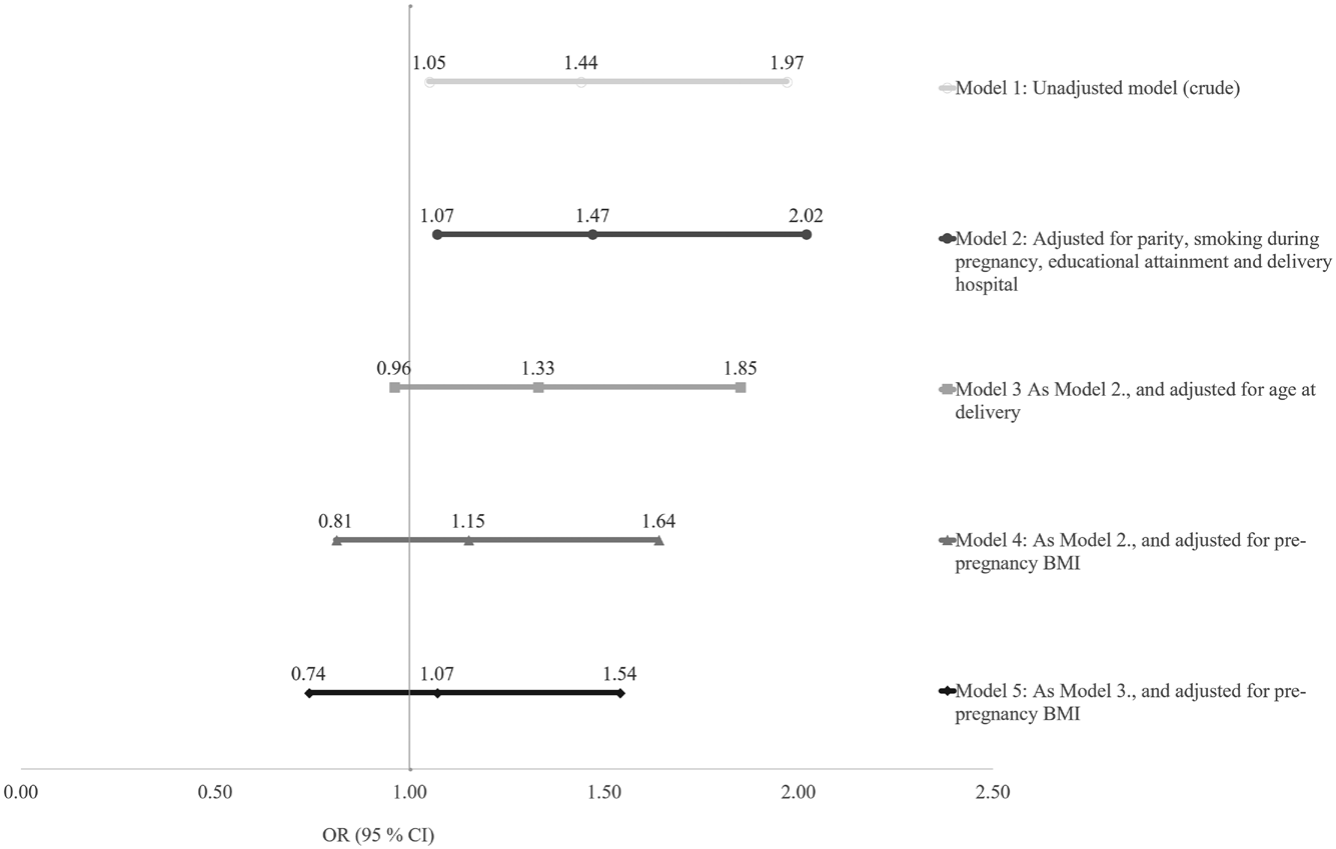
Odds ratios (ORs) for gestational diabetes mellitus (GDM) in women with polycystic ovary syndrome (PCOS) calculated by logistic regression (*N* = 174).

The prevalence of self-reported symptoms and/or prior diagnosis of PCOS was 10.4% among GDM women and 7.4% among non-diabetic women (OR 1.44, 95% CI: 1.05–1.97) (Model 1). The difference remained significant after adjusting for parity, smoking during pregnancy, educational attainment and delivery hospital (Model 2) (OR 1.47, 95% CI: 1.07–2.02), but disappeared after further adjustments for age and pre-pregnancy BMI (OR 1.07, 95% CI: 0.74–1.54) (Model 5), suggesting that PCOS is not an independent risk factor for GDM (Fig. 2). The results remained similar regardless of whether the definition of PCOS was based on symptoms or prior diagnosis. Moreover, the exclusion of the participants with an isolated PCOS symptom from the control group did not affect the results. Further, the results did not change after excluding participants with GDM diagnosed by self-monitoring (*N* = 24).

### Participants’ birth

Participants who were themselves born with a lower birth weight had a higher risk of GDM regardless of the presence of PCOS (Tables 1 and 2). This association was mostly due to slower foetal growth, as indicated by a lower s.d. score for birth weight. No linear association was observed with participant’s own gestational age, but participants born preterm had a two-fold risk of GDM (Table 3).

**Table 1:** Characteristics of participants in each subgroup (*n* = 1941).

Characteristic	Mean (s.d.)/*n* (%)				No. missing
	GDM + PCOS (*n* = 105)	GDM + non-PCOS (*n* = 909)	Non-GDM + PCOS (*n* = 69)	Controls (*n* = 858)	
Participants’ birth^a^					
Birth weight (g)	3323 (570)	3392 (559)	3511 (474)	3471 (505)	7/77/8/58
s.d. score for birth weight	−0.35 (1.16)	−0.14 (1.07)	0.04 (1.00)	0.02 (1.02)	20/192/14/167
Gestational age (weeks)	39.7 (1.7)	39.7 (2.2)	40.3 (1.5)	39.8 (1.8)	17/194/11/159
<37 + 0 weeks (%)	4 (4.5)	46 (6.4)	0	23 (3.3)	17/194/11/159
SGA^b^ (%)	6 (7.1)	24 (3.3)	1 (1.8)	15 (2.2)	20/192/14/167
LGA^c^ (%)	0	21 (2.9)	1 (1.8)	19 (2.7)	20/192/14/167
Participants’ educational attainment^a^					
Basic or less (%)	4 (3.8)	62 (6.8)	1 (1.4)	41 (4.8)	
Secondary (%)	39 (37.1)	437 (48.1)	27 (39.1)	394 (45.9)	
Lower-level tertiary (%)	45 (42.9)	223 (24.5)	19 (27.5)	210 (24.5)	
Upper-level tertiary (%)	17 (16.2)	187 (20.6)	22 (31.9)	213 (24.8)	
Participants’ current characteristics					
Age at delivery (years)	32.0 (5.0)	31.5 (5.4)	29.9 (3.8)	28.8 (5.1)	
Height (cm)	164.9 (5.9)	164.7 (5.8)	166.2 (6.1)	165.3 (5.9)	
Weight (kg)	80.8 (18.6)	75.9 (17.0)	67.8 (14.9)	64.7 (12.3)	
Pre-pregnancy BMI (kg/m^2^)	29.7 (6.6)	27.9 (5.9)	24.5 (5.1)	23.6 (4.1)	
Smoking habit, *n* (%)					1/2/0/1
Before and during pregnancy (%)	16 (15.4)	144 (15.9)	13 (18.8)	127 (14.8)	
Before but not during pregnancy (%)	10 (9.6)	137 (15.1)	8 (11.6)	122 (14.2)	
Not at all (%)	78 (75.0)	623 (68.7)	48 (69.6)	607 (70.8)	
Number of previous pregnancies					
0 (%)	35 (33.3)	272 (29.9)	29 (42.0)	341 (39.7)	
1 (%)	23 (21.9)	246 (27.1)	23 (33.3)	216 (25.2)	
2 (%)	17 (16.2)	153 (16.8)	5 (7.2)	123 (14.3)	
3 or more (%)	30 (28.6)	238 (26.2)	12 (17.4)	178 (20.8)	
Number of previous deliveries					
0 (%)	44 (41.9)	388 (42.7)	38 (55.1)	433 (50.5)	
1 (%)	40 (38.1)	254 (27.9)	18 (26.1)	212 (24.7)	
2 (%)	11 (10.5)	118 (13.0)	8 (11.6)	102 (11.9)	
3 or more (%)	10 (9.5)	149 (16.4)	5 (7.2)	111 (12.9)	
Insulin-treated GDM, *n* (%)	27 (26.0)	149 (16.8)			1/21
Metformin-treated GDM, *n* (%)	4 (3.8)	21 (2.4)			1/21
Diet-treated GDM, *n* (%)	73 (70.2)	718 (83.2)			1/21

^a^As reported by the participants at recruitment in this study; ^b^small for gestational age (<−2 s.d.); ^c^large for gestational age (>+2 s.d.).

GDM, gestational diabetes mellitus; PCOS, polycystic ovary syndrome; s.d., standard deviation.

**Table 2:** Crude and adjusted^a^ odds ratios (ORs) and mean differences in characteristics at birth and present for participants with polycystic ovary syndrome (PCOS) and gestational diabetes mellitus (GDM) or both compared with healthy controls (*n* = 1941).

Characteristic	OR/mean difference (95% CI)					
	GDM + PCOS (*n* = 105)		GDM + non-PCOS (*n* = 909)		Non-GDM + PCOS (*n* = 69)	
	Crude	Adjusted^a^	Crude	Adjusted^a^	Crude	Adjusted^a^
Participants’ birth^b^						
Birth weight (g)	−147 (−255 to −40)	−181 (−299 to −64)	−79 (−131 to −27)	−117 (−174 to −60)	41 (−90 to 172)	47 (−84 to 179)
s.d. score for birth weight	−0.37 (−0.61 to −0.14)	−0.40 (−0.67 to −0.14)	−0.14 (−0.25 to −0.04)	−0.23 (−0.35 to −0.10)	0.03 (−0.26 to 0.31)	0.06 (−0.22 to 0.34)
Gestational age (weeks)	−0.1 (−0.5 to 0.3)	−0.2 (−0.6 to 0.3)	−0.2 (−0.4 to 0.05)	−0.2 (−0.5 to −0.01)	0.5 (0.03–1.0)	0.5 (0.01–0.9)
<37 + 0 weeks	1.43 (0.48–4.22)	1.47 (0.43–4.99)	2.00 (1.20–3.33)	2.24 (1.28–3.92)		
SGA^c^	3.42 (1.29–9.07)	4.08 (1.30–12.6)	1.56 (0.81–3.00)	1.66 (0.81–3.44)	0.84 (0.11–6.44)	0.99 (0.13–7.78)
Participants’ current characteristics						
Age at delivery (years)	3.2 (2.2–4.2)	2.5 (1.6–3.4)	2.7 (2.2–3.2)	2.2 (1.8–2.7)	1.1 (−0.1 to 2.3)	0.9 (−0.1 to 1.9)
Height (cm)	−0.5 (−1.6 to 0.7)	−0.9 (−2.2 to 0.4)	−0.6 (−1.1 to −0.1)	−0.9 (−1.5 to −0.3)	0.9 (−0.5 to 2.4)	0.6 (−0.8 to 2.1)
Weight (kg)	16.1 (13.4–18.8)	15.2 (12.4–17.9)	11.2 (9.8–12.6)	9.9 (8.4–11.3)	3.2 (0.1–6.2)	3.1 (0.0–6.1)
Pre-pregnancy BMI (kg/m^2^)	6.1 (5.2–7.0)	5.8 (4.8–6.7)	4.3 (3.8–4.8)	3.9 (3.4–4.4)	0.9 (−0.1 to 1.9)	1.0 (−0.1 to 2.0)

^a^Linear regression for continuous variables and logistic regression for categorical variables adjusted for participants’ parity, smoking during pregnancy, educational attainment, delivery hospital, age and pre-pregnancy BMI; ^b^as reported by the participants at recruitment in this study; ^c^small for gestational age (<−2 s.d.).

GDM, gestational diabetes mellitus; PCOS, polycystic ovary syndrome; s.d., standard deviation.

**Table 3:** Crude and adjusted odds ratios (ORs) for risk factors of gestational diabetes mellitus (GDM) calculated by logistic regression (*n* = 2212).

Risk factor	Crude OR	Adjusted OR^a^
Own preterm birth^b^	2.10 (1.27–3.48)	2.32 (1.34–4.03)
PCOS	1.44 (1.05–1.97)	1.07 (0.74–1.54)
BMI ≥25 kg/m^2^	4.87 (4.07–5.84)	4.22 (3.44–5.18)
BMI ≥30 kg/m^2^	5.47 (4.26–7.02)	5.14 (3.87–6.83)
Age ≥35 years	2.34 (1.90–2.88)	2.58 (1.98–3.38)
Participant’s mother’s GDM	2.43 (1.62–3.65)	3.05 (1.93–4.82)
Participant’s mother’s T2D	2.64 (1.92–3.62)	1.85 (1.30–2.64)
Participant’s father’s T2D	1.77 (1.37–2.28)	1.37 (1.03–1.83)

^a^Logistic regression for categorical variables adjusted for participants’ parity, smoking during pregnancy, educational attainment, delivery hospital, age and pre-pregnancy BMI; ^b^participant born <37 + 0 weeks of gestation.

PCOS, polycystic ovary syndrome; T2D, type 2 diabetes.

Participants in the non-GDM + PCOS group were born, on average, 0.5 weeks later than the controls (Tables 1 and 2). Participants with both GDM + PCOS were born three times as often SGA than the controls (*P* = 0.013). This finding was not related to participants’ pre-pregnancy BMI.

### Participants’ educational attainment and health

Participants with GDM, regardless of PCOS status, were less educated in comparison to controls (*P* = 0.012). Lower educational attainment correlated with higher pre-pregnancy BMI (*P* < 0.001). In addition, participants with GDM, regardless of PCOS status, were significantly older (*P* < 0.001) and more often had three or more previous pregnancies (*P* = 0.001) and deliveries (*P* = 0.014) than the controls (Table 1).

**Figure 3:**
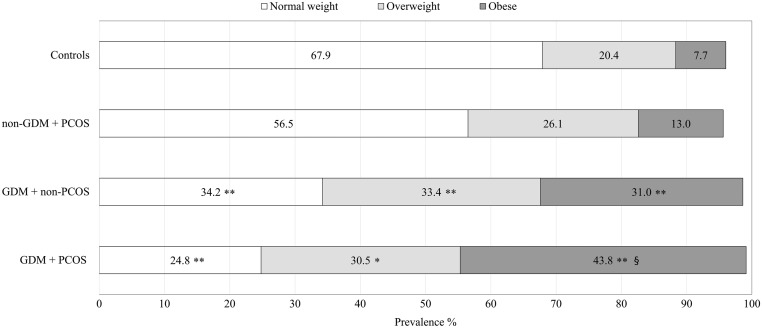
Prevalence of normal weight, overweight and obese women in the subgroups. *P* values for difference between each subgroup and the control group: *<0.05; **<0.001. *P* value for difference between GDM + PCOS group and GDM + non-PCOS group: ^§^0.009. Normal weight BMI: 18.5–24.9 kg/m^2^, overweight BMI: 25.0–29.9 kg/m^2^ and obese BMI: ≥30.0 kg/m^2^.

Participants with GDM were shorter and had higher pre-pregnancy BMI than controls (Tables 1 and 2). A difference in height was present in both the GDM + PCOS (−0.5 cm) and GDM + non-PCOS (−0.6 cm) groups but was statistically significant only in the latter. Pre-pregnancy BMI was significantly higher in the GDM + PCOS group compared to the other three groups, and participants in this group were more likely to be obese (Fig. 3). The results did not change after adjusting for participants’ parity, smoking during pregnancy, educational attainment, delivery hospital and age (Tables 1 and 2).

### Medical histories of participants’ parents’

The participant’s risk for GDM was doubled if her mother had a history of GDM or T2D (Tables 3, 4 and 5). If the participant’s father had a history of T2D, the participant’s risk for GDM was 1.4-fold. If either of a participant’s parents had a history of hypertension, her risk for GDM was 1.5-fold. The result was, however, non-significant after adjusting for pre-pregnancy BMI. Parental diabetes and other cardiovascular morbidities were not associated with the presence of PCOS.

**Table 4:** Participants’ parents’ education and medical histories categorised by subgroup (*n* = 1941).

Characteristic	Mean (s.d.)/*n* (%)				No. missing
	GDM + PCOS (*n* = 105)	GDM + non-PCOS (*n* = 909)	Non-GDM + PCOS (*n* = 69)	Controls (*n* = 858)	
Education of participants’ parents^a^					
Highest education of either parent					1/14/0/6
Basic or less (%)	25 (24.0)	188 (21.0)	10 (14.5)	142 (16.7)	
Secondary (%)	61 (58.7)	526 (58.8)	42 (60.9)	465 (54.3)	
Lower-level tertiary (%)	6 (5.8)	61 (6.8)	7 (10.1)	86 (10.1)	
Upper-level tertiary (%)	12 (11.5)	120 (13.4)	10 (14.5)	161 (18.9)	
Medical history of participants’ parents^a^					
Pregnancy history of participants’ mothers					
GDM^b^ (%)	2 (2.2)	25 (3.1)	1 (1.5)	13 (1.6)	4/92/14/55
GDM at any pregnancy (%)	6 (5.9)	80 (9.3)	2 (3.2)	32 (4.0)	3/47/7/48
Pre-eclampsia^b^ (%)	7 (7.9)	46 (5.4)	1 (1.5)	35 (4.4)	16/75/4/64
Medical history of participants’ mothers					
Diabetes mellitus (%)	16 (15.5)	136 (15.4)	6 (9.5)	52 (6.3)	2/25/6/27
Hypertension (%)	27 (27.0)	255 (29.0)	13 (20.3)	180 (21.8)	5/31/5/34
Myocardial infarction (%)	2 (2.0)	13 (1.5)	2 (3.2)	15 (1.8)	3/26/6/27
Stroke (%)	1 (1.0)	26 (2.9)	4 (6.3)	14 (1.7)	4/23/5/28
Medical history of participants’ fathers					
Diabetes mellitus (%)	25 (24.8)	159 (18.5)	8 (13.1)	99 (12.0)	4/48/8/34
Hypertension (%)	28 (28.9)	247 (29.4)	18 (30.0)	177 (21.8)	8/70/9/45
Myocardial infarction (%)	11 (11.2)	91 (10.6)	5 (8.3)	79 (9.6)	7/50/9/36
Stroke (%)	3 (3.1)	47 (5.5)	2 (3.4)	30 (3.6)	7/47/10/36

^a^As reported by the participants at recruitment in this study; ^b^during the pregnancy when the study participant was born.

GDM, gestational diabetes mellitus; PCOS, polycystic ovary syndrome.

**Table 5:** Crude and adjusted^a^ odds ratios (ORs) of the medical histories of the parents of women with polycystic ovary syndrome (PCOS) and gestational diabetes mellitus (GDM) or both compared with healthy controls (*n* = 1941).

Characteristic	OR (95% CI)					
	GDM + PCOS (*n* = 105)		GDM + non-PCOS (*n* = 909)		Non-GDM + PCOS (*n* = 69)	
	Crude	Adjusted^a^	Crude	Adjusted^a^	Crude	Adjusted^a^
Pregnancy history of participants’ mothers^b^						
GDM^c^	1.37 (0.30–6.15)	1.80 (0.34–9.44)	1.92 (0.97–3.78)	1.86 (0.89–3.91)	0.95 (0.12–7.38)	0.98 (0.12–7.68)
GDM at any pregnancy	1.52 (0.62–3.73)	2.30 (0.82–6.48)	2.49 (1.63–3.79)	3.25 (2.04–5.18)	0.81 (0.19–3.46)	0.95 (0.22–4.15)
Pre-eclampsia^c^	1.85 (0.80–4.30)	1.48 (0.58–3.75)	1.27 (0.81–1.99)	0.94 (0.57–1.55)	0.34 (0.05–2.51)	0.27 (0.04–2.00)
Medical history of participants’ mothers^b^						
Diabetes mellitus	2.76 (1.51–5.03)	2.16 (1.06–4.40)	2.72 (1.95–3.81)	1.87 (1.29–2.72)	1.58 (0.65–3.83)	1.41 (0.57–3.46)
Hypertension	1.32 (0.83–2.12)	1.27 (0.74–2.16)	1.46 (1.13–1.83)	1.23 (0.96–1.57)	0.91 (0.49–1.71)	0.90 (0.48–1.72)
Myocardial infarction	1.09 (0.25–4.83)	0.39 (0.06–2.59)	0.81 (0.38–1.72)	0.43 (0.18–1.04)	1.78 (0.40–7.98)	1.27 (0.27–5.96)
Stroke	0.58 (0.08–4.48)	0.78 (0.09–6.79)	1.76 (0.91–3.40)	1.36 (0.65–2.83)	3.89 (1.24–12.2)	3.74 (1.16–12.0)
Medical history of participants’ fathers^b^						
Diabetes mellitus	2.41 (1.46–3.97)	1.76 (0.99–3.11)	1.66 (1.26–2.18)	1.26 (0.94–1.71)	1.11 (0.51–2.39)	1.03 (0.47–2.26)
Hypertension	1.46 (0.91–2.33)	1.36 (0.80–2.30)	1.50 (1.20–1.87)	1.22 (0.95–1.57)	1.54 (0.87–2.74)	1.46 (0.81–2.62)
Myocardial infarction	1.19 (0.61–2.32)	0.80 (0.36–1.78)	1.11 (0.81–1.53)	0.86 (0.60–1.23)	0.86 (0.33–2.20)	0.88 (0.34–2.28)
Stroke	0.83 (0.25–2.78)	0.82 (0.20–3.43)	1.52 (0.95–2.43)	1.50 (0.88–2.54)	0.93 (0.22–3.97)	1.05 (0.24–4.56)

^a^Logistic regression for categorical variables adjusted for participants’ parity, smoking during pregnancy, educational attainment, delivery hospital, age and pre-pregnancy BMI; ^b^as reported by the participants at recruitment in this study; ^c^during the pregnancy when the study participant was born.

### Risk factors for GDM

In the multivariate regression analyses, the most significant parameters independently associated with participant’s GDM were adiposity, age (≥35 years), own preterm birth, participant’s mother’s history of GDM or either parent’s history of T2D (Table 3). In total, 27 (26.0%) of the women with GDM + PCOS and 149 (16.8%) of the women with GDM + non-PCOS were treated with insulin during pregnancy (*P* = 0.022) (Table 1). The difference between the groups was related to higher BMI (*P* = 0.056).

## Discussion

In this case–control study, self-reported symptoms and/or prior diagnosis of PCOS was not an independent risk factor for GDM. Indeed, the prevalence of GDM was higher in women with PCOS than in women without PCOS, but the difference was mediated by maternal adiposity and higher age rather than by the syndrome itself. As expected, GDM was predicted by a family history of GDM and T2D. This finding seemed to be similar for participants with GDM regardless of PCOS status.

Several studies have shown that PCOS is an independent risk factor for GDM (2, 15, 16, 17, 18). In the present study, however, we found that obesity is the most important risk factor for GDM in women with PCOS. In line with this result, in a previous Finnish study, overweight was the strongest predictor for GDM, although PCOS slightly increased the risk (19). Furthermore, other studies did not find an increased risk for GDM in non-obese women with PCOS or when participants were matched according to BMI and age (20, 21, 22). Possible reasons for these discordant results may be differences in diagnostic criteria and heterogeneous study populations. In the present study, PCOS women without GDM were significantly leaner than women with GDM, and their mean BMI was similar to the BMI of the controls, which may explain the findings. This result is also in keeping with the findings of a recent large Finnish cohort study in which only overweight and obese women with PCOS were at risk for subsequent T2D (31). These results suggest that obese women with PCOS are at substantial risk for GDM and should, therefore, be recognised and counselled as a high-risk group before or during the early stages of pregnancy to achieve more efficient therapeutic interventions. Inversely, the present findings challenge the utility of a routine OGTT during early pregnancy in women with PCOS in the absence of other risk factors.

In addition to overweight and obesity, age and a family history of diabetes were significant risk factors for GDM, as expected and in line with the results of previous studies (32, 33, 34). In particular, if a participant’s mother had a history of GDM or T2D, the participant’s risk for GDM increased two- to three-fold, in accordance with previous data (35, 36, 37). Also, father’s history of T2D increased the participant’s risk of GDM. In the present study, the risk remained elevated even after adjusting for participants’ BMI, suggesting that genetic factors may play a strong role in the development of GDM in addition to lifestyle and environmental conditions.

Interestingly, 7% of participants with GDM + PCOS were born SGA, which was three times that of the control group. Additionally, the participants with GDM, regardless of their PCOS status, were twice as likely to be born preterm. These women were also slightly shorter than controls, in line with previous reports showing a 1.9–3.0 cm height difference, which may be explained by genetic, hormonal or nutritional issues as well as by socio-economic issues (38, 39, 40). Due to the relatively small sample sizes, our results must be interpreted with caution, but they are consistent with earlier observations that low birth weight and preterm birth increase an individual’s risk of GDM (41, 42). Preterm birth is also a well-established risk factor for T2D and related cardiometabolic risk factors (43, 44, 45). Moreover, the catch-up growth of SGA children is associated with insulin resistance in childhood (46, 47).

To the best of our knowledge, this is the first study comparing women with GDM, PCOS or both with controls in a population-based data set. Importantly, in this large, well-defined nationwide and homogenous study population (99.8% of Caucasian origin), we were able to take into account most of the potential confounders. An important limitation of the study is that the definition of PCOS was based on a questionnaire, since the study setting did not allow a clinical examination of the participants and the diagnosis of PCOS could not be made during pregnancy. Though hirsutism is subjective and may easily be over-reported, we have previously shown that self-reported isolated hirsutism does correlate with increased androgen secretion and that self-reported oligomenorrhoea and hirsutism can identify women with the typical endocrine and metabolic profiles of PCOS (25, 26). The participants’ reports of prior PCOS diagnoses made by a physician could not be verified from the medical records, which may increase the heterogeneity of the study population and the presence of mild states of PCOS. However, the results were congruent regardless of whether the definition of PCOS was based on symptoms or self-report. Finally, all women with PCOS participating in the present study had become pregnant and delivered, which may also indicate a milder disorder and a more favourable metabolic profile compared with women who did not become pregnant. This issue may have decreased the incidence of GDM in the subgroup of women with PCOS.

We conclude that PCOS is not an independent risk factor for GDM and that the increased risk of GDM in women with PCOS is mainly related to adiposity. In particular, obese women with PCOS are at substantial risk for GDM and often need insulin treatment during pregnancy. Therefore, they should be recognised and counselled as a high-risk group before or during the early stages of pregnancy to achieve more efficient therapeutic interventions. Inversely, the utility of a routine early OGTT during pregnancy in PCOS women in the absence of other risk factors should be reconsidered. Last, a family history of GDM or T2D, elevated maternal age over 35 years and own preterm birth should be noted as risk factors for GDM. These actions could help identify individuals at risk for GDM and focus on preventive measures.

## Declaration of interest

The authors declare that there is no conflict of interest that could be perceived as prejudicing the impartiality of the research reported.

## Funding

This work was supported by Academy of Finland (grant numbers 127437, 128929, 129306, 130326, 134791, 274794); Foundation for Pediatric Research; Diabetes Research Foundation; Juho Vainio Foundation; Novo Nordisk Foundation; Signe and Ane Gyllenberg Foundation; Sigrid Jusélius Foundation and Yrjö Jahnsson Foundation.

## Authors’ contribution statement

M V, H L, J G E, M G, E K and L C M-P designed the FinnGeDi study and contributed to data collection. M V, E K, S M and L C M-P designed the present study. A B participated in data management. S M participated in data collection, cleaning and analysis. S M and E K performed data analysis and M V, H L, J G E, H I, M G, A B and L C M-P contributed to its interpretation. S M wrote the first draft of the manuscript and L C M-P, M V and E K reviewed, completed and supervised the manuscript writing. All authors read and approved the final manuscript.
